# Effects of excitatory transcranial magnetic stimulation over the different cerebral hemispheres dorsolateral prefrontal cortex for post-stroke cognitive impairment: a systematic review and meta-analysis

**DOI:** 10.3389/fnins.2023.1102311

**Published:** 2023-05-16

**Authors:** Kaiyue Han, Jiajie Liu, Zhiqing Tang, Wenlong Su, Ying Liu, Haitao Lu, Hao Zhang

**Affiliations:** ^1^School of Rehabilitation, Capital Medical University, Beijing, China; ^2^China Rehabilitation Research Center, Beijing Bo'ai Hospital, Beijing, China; ^3^University of Health and Rehabilitation Sciences, Qingdao, China; ^4^Cheeloo College of Medicine, Shandong University, Jinan, China

**Keywords:** stroke, transcranial magnetic stimulation, dorsolateral prefrontal cortex, cognitive function, meta-analysis

## Abstract

**Background:**

Post-stroke cognitive impairment (PSCI) is a significant health concern. Transcranial magnetic stimulation (TMS) is considered a promising rehabilitation therapy for improving cognition, and the effects of excitatory TMS on PSCI have received much attention in recent years. However, the effects of different cerebral hemispheres on excitatory TMS treatment of cognitive impairment have not been studied. This review aimed to study the effects of excitatory TMS over the dorsolateral prefrontal cortex (DLPFC) of different cerebral hemispheres on the cognitive function of patients with PSCI.

**Methods:**

Literature published in PubMed, Web of Science, Embase, Cochrane Library, Scopus, and Wiley from inception to September 30, 2022, were searched. Two researchers independently performed literature screening, data extraction, and quality assessment. Furthermore, we conducted a meta-analysis using RevMan software (version 5.4) and rated the strength of evidence using GRADEpro.

**Results:**

A total of 19 studies were included in this meta-analysis. The results showed that excitatory TMS over the left hemisphere DLPFC was significantly better in improving global cognition (SMD = 2.26, 95% CI 1.67–2.86, *P* < 0.00001; vs. SMD = 2.53, 95% CI 1.86–3.20, *P* < 0.00001), memory (SMD = 1.29, 95% CI 0.72–1.87, *P* < 0.0001), attention (SMD = 2.32, 95% CI 1.64–3.01, *P* < 0.00001), executive (SMD = 0.64, 95% CI 0.21–1.07, *P* = 0.004), P300 latency (SMD = 2.69, 95% CI 2.13–3.25, *P* < 0.00001), and depression (SMD = 0.95, 95% CI 0.26–1.63, *P* = 0.007) than that of the control group, but the effect on improving activities of daily living (ADL) was unclear (*P* = 0.03 vs. *P* = 0.17). Subgroup analysis further showed that excitatory TMS over the right hemisphere DLPFC was effective in improving the global cognition of PSCI patients (*P* < 0.00001), but the stimulation effect over the ipsilateral hemisphere DLPFC was unclear (*P* = 0.11 vs. *P* = 0.003). Additionally, excitatory TMS over the ipsilateral hemisphere DLPFC showed no statistical difference in improving ADL between the two groups (*P* = 0.25).

**Conclusions:**

Compared to other hemispheric sides, excitatory TMS over the left hemisphere DLPFC was a more effective stimulation area, which can significantly improved the global cognitive function, memory, attention, executive, P300 latency, and depression in patients with PSCI. There was no apparent therapeutic effect on improving activities of daily living (ADL). In the future, more randomized controlled trials with large-sample, high quality, and follow-up are necessary to explore a usable protocol further.

**Systematic review registration:**

https://www.crd.york.ac.uk/PROSPERO/, identifier: CRD42022369096.

## 1. Introduction

Stroke, a cerebrovascular disease with high mortality and disability rates, can expose survivors to various dysfunctions. Cognitive impairment is a common post-stroke complication with an incidence of 20–80% (Huang et al., 2022). Notably, post-stroke cognitive impairment (PSCI) refers to a series of syndromes that meet the diagnostic criteria of cognitive dysfunction within 6 months after the clinical event of stroke (Rost et al., 2022), mainly manifested in memory decline, inattention, and executive dysfunction. According to epidemiological data, 17–92% of stroke patients experience cognitive impairment within 3 months after onset (Snyder et al., 2015), severely impacting activities of daily living (ADL) and quality of survival.

Traditional cognitive rehabilitation has primarily improved function through pharmacological therapy or compensatory strategies. Unfortunately, evidence for such effects remains limited and clinical efficacy is poor (Zhao et al., 2021). In recent years, transcranial magnetic stimulation (TMS), a non-invasive neuromodulation technique, is applied to the cerebral cortex with a pulsed magnetic field to induce changes in its local or distal neural activity (Kobayashi and Pascual-Leone, 2003). According to the different modulations of cortical excitability, TMS can be divided into excitatory and inhibitory types (Gilio et al., 2007). Excitatory TMS includes high-frequency rTMS (HF-rTMS) and intermittent theta burst stimulation (iTBS), which can promote neuronal activity (Wang et al., 2018). In contrast, inhibitory TMS includes low-frequency rTMS (LF-rTMS) and continuous theta burst stimulation (cTBS), which can inhibit neuronal activity (Li et al., 2021a).

From literature reviews, most studies performed excitatory TMS treatment over the dorsolateral prefrontal cortex (DLPFC) of patients with PSCI. It is well known that DLPFC is closely related to the process of cognitive control and plays an important role in the recovery of memory, attention, execution, and other cognitive functions after stroke (Chen et al., 2013; Webler et al., 2022). Studies have shown that Excitatory TMS over the DLPFC can affect intracerebral metabolism and increase cortical excitability, altering neuronal activity in the target cortical area and functional connectivity between brain networks to improve cognitive function in patients with PSCI (Wilson et al., 2018; Wu et al., 2021).

DLPFC is a relatively large area (Siebner and Rothwell, 2003), and the application sites of excitatory TMS in the treatment of cognitive impairment are different. Four studies (Ding et al., 2019; Wang et al., 2019, 2021; Cha et al., 2022) applied high-frequency rTMS over the ipsilateral hemisphere DLPFC in patients with PSCI, while Bie and Wang (2011) over the right DLPFC, and all of these studies have reported certain positive effects in improving the cognition of patients. In recent years, we have found that more studies have focused on excitatory TMS treatment over the left hemisphere DLPFC for PSCI patients to improve cognition. However, the difference between the left and right hemispheres is an important effect factor, and there is a lack of research on stimulating DLPFC over different cerebral hemispheres to treat cognitive impairment with TMS. Therefore, the application site of excitatory TMS remains controversial (Yang et al., 2015b). Based on this, the aim of this study was to analyze the effects of excitatory TMS over the DLPFC of different cerebral hemispheres on cognitive function in patients with PSCI.

## 2. Methods

This review was conducted according to the Preferred Reporting Items for Systematic Reviews and Meta-Analyses (PRISMA) (Moher et al., 2015). It was registered in the International Prospective Register of Systematic Reviews (CRD42022369096).

### 2.1. Search strategy

Two investigators independently performed the literature published in PubMed, Web of Science, Embase, Cochrane Library, Scopus, and Wiley from inception to September 30, 2022. Additionally, we manually searched all reference lists of the selected articles and related review articles, and we used the same search terms in Google Scholar to perform additional searches. We used the search terms “transcranial magnetic stimulation,” “stroke,” and “cognitive function,” or their synonyms. The detailed search strategy is provided in Supplementary Table S1.

### 2.2. Selection of studies

Studies were included in this study if they met the following criteria: (1) population: adult patients (≥18 years) diagnosed with stroke and cognitive dysfunction; (2) intervention: HF-rTMS or iTBS over the DLPFC, with or without conventional rehabilitation; (3) control: sham stimulation or placebo or blank control, with or without conventional rehabilitation; (4) results: measures that evaluated the global cognition or memory or attention or execution; (5) study type: randomized controlled trials (RCTs) or prospective controlled trials (PCTs); (6) language: English and Chinese.

### 2.3. Data collection and extraction

Two researchers (HKY, LJJ) independently screened the literature, extracted information, and cross-checked it. In case of disagreement, a third researcher (TZQ) reviewed until a consensus was reached. For every study, we extracted the following information: the name of the first author, the year of publication, country, dysfunction diagnosis, sample size, patient characteristics (gender, age, onset time of stroke, and education), intervention protocol (site of stimulation, type of TMS, frequency, intensity, and duration), control condition, outcome measures, follow-up, drop-out rate, and PEDro score. We emailed the authors for questionable or incomplete data to clarify or add the missing information. Only data immediately after the intervention were extracted for studies that included post-intervention and follow-up data. If the results were only presented graphically, we used GetData Graph Digitizer 2.20 to extract the required data (Zhang et al., 2017).

### 2.4. Risk of bias and quality assessment

Two reviewers (HKY, LJJ) independently assessed the bias of the included studies according to the Cochrane Handbook for Systematic Reviews of Interventions (Higgins et al., 2011), and disagreements were resolved by discussing with the third reviewer (TZQ). The assessment items included selection bias, performance bias, detection bias, attrition bias, reporting bias, and other biases. Each item was rated as “high,” “low,” or “unclear.”

The PEDro scale (consisting of 11 items) was used to assess the methodological quality of the included studies, and studies with a score of <6 were considered low-quality (Cashin and McAuley, 2020). Furthermore, we used the online GRADEpro to assess the quality of evidence for pooled results in this meta-analysis, including the risk of bias, inconsistency, indirectness, imprecision, and publication bias (Cui et al., 2019).

### 2.5. Statistical analysis

We used RevMan 5.4 to perform the meta-analysis. The Mini-mental state examination (MMSE) and Montreal cognitive assessment (MoCA) were used to assess patients' global cognitive function. The Rivermead behavioral memory test (RBMT) was used to assess memory. The Trail Making Test (TMT), Digit Symbol Test (DST), and Digital Span Test (DS) were used to evaluate attention. The Stroop Color and Word test (SCWT) was used to assess executive function. The Modified Barthel Index (MBI) and independent function measure (FIM) were used to assess the ADL. The event-related potential (ERP) P300 was used to evaluate cognitive deterioration, and Beck's Depression Inventory (BDI) was used to assess depression. Since all data were continuous information and measuring the same outcome using different scales, we selected Standardized Mean Difference (SMD) with 95% confidence intervals (CIs). We used the Cochrane Q statistic to qualitatively determine whether heterogeneity existed among the included studies (test level α = 0.05), while the *I*^2^ statistic to assess the magnitude of heterogeneity quantitatively. If *P* ≥ 0.1 and *I*^2^ ≤ 50%, the heterogeneity was considered insignificant, and we selected the fixed-effect (FE) model. Conversely, we selected the random-effect (RE) model and performed a subgroup analysis and sensitivity analysis to identify factors that might cause heterogeneity. Descriptive analysis was used if the source of heterogeneity could not ultimately be determined.

## 3. Results

### 3.1. Study selection

**Figure 1:**
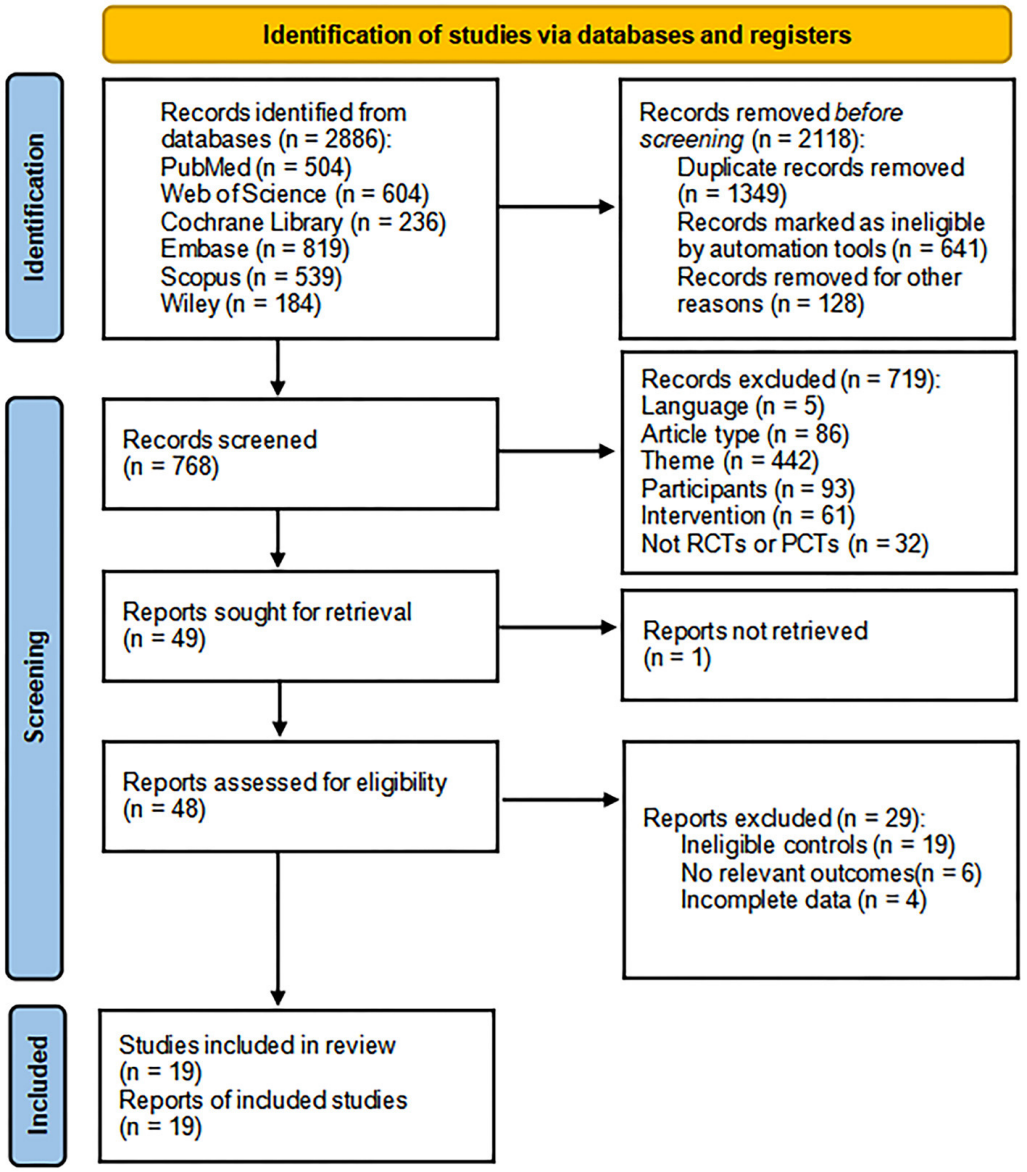
PRISMA flowchart of study selection.

We initially retrieved 2,886 articles from 6 databases, and tools removed 2,118 articles before the screening. The 719 articles that did not meet the criteria were removed after reading the title and abstract, and one article was not retrieved. After that, the remaining 48 articles were read in full text, of which 19 articles had no eligible controls, 6 articles had no relevant outcomes, and 4 articles could not get complete data. Finally, 19 articles were included in this meta-analysis (Kim et al., 2010; Bie and Wang, 2011; Liu et al., 2017, 2020; Zheng et al., 2017, 2020; Yin et al., 2018, 2020; Ding et al., 2019; Luo and Yu, 2019; Wang et al., 2019, 2021; Zhang and Zou, 2019; Li et al., 2020, 2022; Tsai et al., 2020; Zhang et al., 2020, 2022; Cha et al., 2022) (Figure 1).

### 3.2. Study characteristics

A total of 19 studies, 17 randomized controlled studies, and 2 prospective controlled studies were included in this meta-analysis. The mean age range of the subjects was 49.07 ± 9.26 (Zhang et al., 2020) to 66.80 ± 17.20 years (Kim et al., 2010), the mean onset time of stroke was 2.73 ± 1.26 (Zheng et al., 2017) to 33.27 ± 26.40 months (Tsai et al., 2020), and the mean years of education was 7.13 ± 4.05 (Ding et al., 2019) to 14.00 ± 2.80 years (Tsai et al., 2020). Regarding the diagnosis of dysfunction, participants in fourteen, one, two, one, and one studies were diagnosed with post-stroke cognitive impairment (Kim et al., 2010; Yin et al., 2018, 2020; Ding et al., 2019; Luo and Yu, 2019; Wang et al., 2019, 2021; Zhang and Zou, 2019; Li et al., 2020, 2022; Tsai et al., 2020; Zheng et al., 2020; Cha et al., 2022; Zhang et al., 2022), post-stroke mild cognitive impairment (Bie and Wang, 2011), post-stroke vascular cognitive impairment (Zheng et al., 2017; Zhang et al., 2020), post-stroke attention impairment (Liu et al., 2020), and post-stroke executive impairment (Liu et al., 2017), respectively. Regarding the stimulation type, 18 studies used HF-rTMS (Kim et al., 2010; Bie and Wang, 2011; Liu et al., 2017, 2020; Zheng et al., 2017, 2020; Yin et al., 2018, 2020; Ding et al., 2019; Luo and Yu, 2019; Wang et al., 2019, 2021; Zhang and Zou, 2019; Li et al., 2020; Tsai et al., 2020; Zhang et al., 2020, 2022; Cha et al., 2022) and 2 studies (Tsai et al., 2020; Li et al., 2022) used iTBS. Regarding the stimulation side of the different cerebral hemispheres, fourteen, one, and four studies selected the left DLPFC (Kim et al., 2010; Liu et al., 2017, 2020; Zheng et al., 2017, 2020; Yin et al., 2018, 2020; Luo and Yu, 2019; Zhang and Zou, 2019; Li et al., 2020, 2022; Tsai et al., 2020; Zhang et al., 2020, 2022), right DLPFC (Bie and Wang, 2011), and ipsilateral DLPFC (Ding et al., 2019; Wang et al., 2019, 2021; Cha et al., 2022), respectively. Regarding the stimulation intensity, eleven, two, three, and one studies were set at 80% (Kim et al., 2010; Bie and Wang, 2011; Zheng et al., 2017, 2020; Yin et al., 2018, 2020; Zhang and Zou, 2019; Tsai et al., 2020; Zhang et al., 2020, 2022; Wang et al., 2021), 90% (Liu et al., 2017, 2020), 100% (Li et al., 2020, 2022; Cha et al., 2022), and 110% (Ding et al., 2019) of the resting motor threshold (RMT), respectively. In regards to the stimulation duration, six, two, eight, one, and two studies performed TMS treatment for 2 weeks (Kim et al., 2010; Bie and Wang, 2011; Ding et al., 2019; Tsai et al., 2020; Cha et al., 2022; Li et al., 2022), 3 weeks (Luo and Yu, 2019; Li et al., 2020), 4 weeks (Liu et al., 2017, 2020; Yin et al., 2018, 2020; Wang et al., 2019; Zhang and Zou, 2019; Zheng et al., 2020; Zhang et al., 2022), 6 weeks (Zheng et al., 2017), and 8 weeks (Zhang et al., 2020; Wang et al., 2021), respectively. Furthermore, only three studies performed follow-up assessments (Bie and Wang, 2011; Ding et al., 2019; Cha et al., 2022). The characteristics of the included studies are detailed in Supplementary Table S2.

### 3.3. Risk of bias and quality assessment

**Figure 2:**
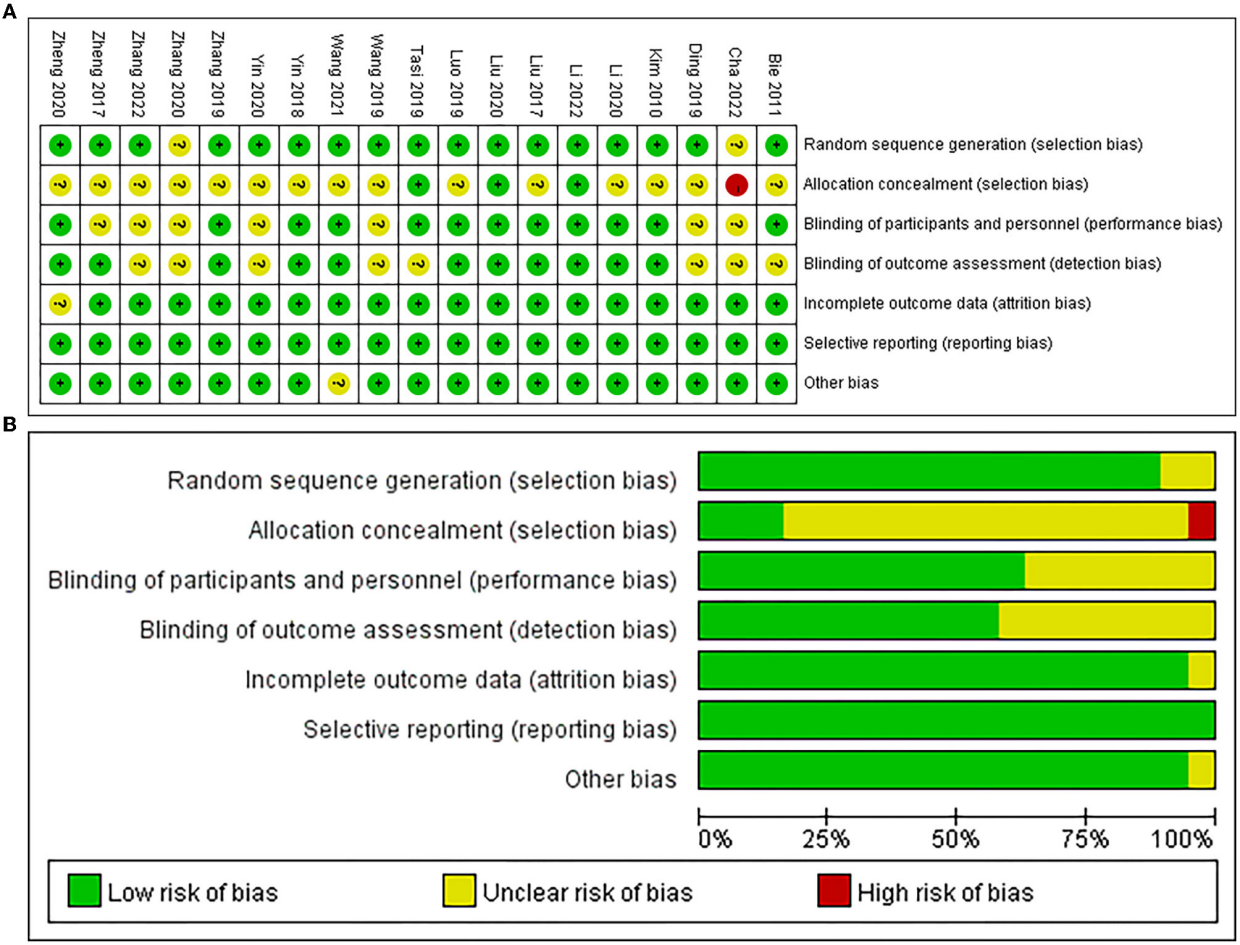
Results from the Cochrane risk of bias (ROB) tool. **(A)** ROB graph, **(B)** ROB summary.

Among the 19 studies included in this meta-analysis, 17 studies performed randomization (Kim et al., 2010; Bie and Wang, 2011; Liu et al., 2017, 2020; Zheng et al., 2017, 2020; Yin et al., 2018; Ding et al., 2019; Luo and Yu, 2019; Wang et al., 2019, 2021; Zhang and Zou, 2019; Li et al., 2020, 2022; Tsai et al., 2020; Zhang et al., 2022), 3 studies performed allocation concealment (Liu et al., 2020; Tsai et al., 2020; Li et al., 2022), 12 studies were blinded to participants and personnel (Kim et al., 2010; Bie and Wang, 2011; Liu et al., 2017, 2020; Yin et al., 2018; Luo and Yu, 2019; Zhang and Zou, 2019; Li et al., 2020, 2022; Tsai et al., 2020; Zheng et al., 2020; Wang et al., 2021), and 11 studies were blinded to assessors (Kim et al., 2010; Liu et al., 2017, 2020; Zheng et al., 2017, 2020; Yin et al., 2018; Luo and Yu, 2019; Zhang and Zou, 2019; Li et al., 2020, 2022; Wang et al., 2021). Additionally, one study reported attrition bias (Zheng et al., 2020) and the other study reported other bias (Wang et al., 2021), respectively, and all studies had no reporting bias (Figure 2).

The PEDro scale demonstrated that 12 studies were of excellent quality (Kim et al., 2010; Liu et al., 2017, 2020; Yin et al., 2018; Ding et al., 2019; Luo and Yu, 2019; Zhang and Zou, 2019; Li et al., 2020, 2022; Tsai et al., 2020; Zheng et al., 2020; Wang et al., 2021) and 7 studies were of good quality (Bie and Wang, 2011; Zheng et al., 2017; Wang et al., 2019; Yin et al., 2020; Zhang et al., 2020, 2022; Cha et al., 2022) in this meta-analysis. For global cognitive function, the GRADE ratings (Zhang et al., 2018) indicated the reliability of excitatory TMS for improving global cognition were both “moderate” using the MMSE and MoCA as outcome measures, respectively (Table 1).

**Table 1:** Summary of the GRADEpro.

**Question:** Effects of excitatory transcranial magnetic stimulation (TMS) over the dorsolateral prefrontal cortex (DLPFC) of different cerebral hemispheres in the global cognition for patients with post-stroke cognitive impairment (PSCI). **Setting:** Hospitals. **Intervention:** Excitatory TMS on the DLPFC, with or without conventional rehabilitation. **Comparison:** Sham stimulation or placebo or blank control, with or without conventional rehabilitation.				
**Outcome measure**	**No of studies**	**No of the participants**	**Anticipated absolute effects** ^*^ **(95% CI)**	**certainty of the evidence (GRADE)**
MMSE	6	247	SMD 1.93 higher (1.38 lower to 2.47 higher)	⊕⊕⊕○ Moderate^a^
MoCA	9	387	SMD 2.32 higher (1.55 lower to 3.10 higher)	⊕⊕⊕○ Moderate^b^
**Certainty of the evidence (GRADE)** High quality: We are very confident that future research lies close to the estimate of effect. Moderate quality: We are moderately confident in the effect estimate. Future research is likely to be close to the estimate of the effect, but there is a possibility that it may change the estimate. Low quality: Our confidence in the effect estimate is limited. Future research may be substantially different from the estimate of the effect and likely to change the estimate. Very low quality: We are very uncertain about the estimate of effect.				

* The risk in the intervention group (and its 95% CI) is based on the assumed risk in the comparison group and the relative effect of the intervention (and its 95% CI).

CI, confidence interval; MMSE, the Mini-Mental State Examination; SMD, standardized mean difference; MoCA, the Montreal Cognitive Assessment.

a Most of the RCTs were low quality with an inadequate level of blinding and unclear risk of concealment of allocation.

b The statistical test for heterogeneity showed that large variation (*I*^2^ > 50%) existed in point estimates due to the among study differences.

### 3.4. Effects of excitatory TMS over the DLPFC in patients with PSCI

#### 3.4.1. Global cognition

**Figure 3:**
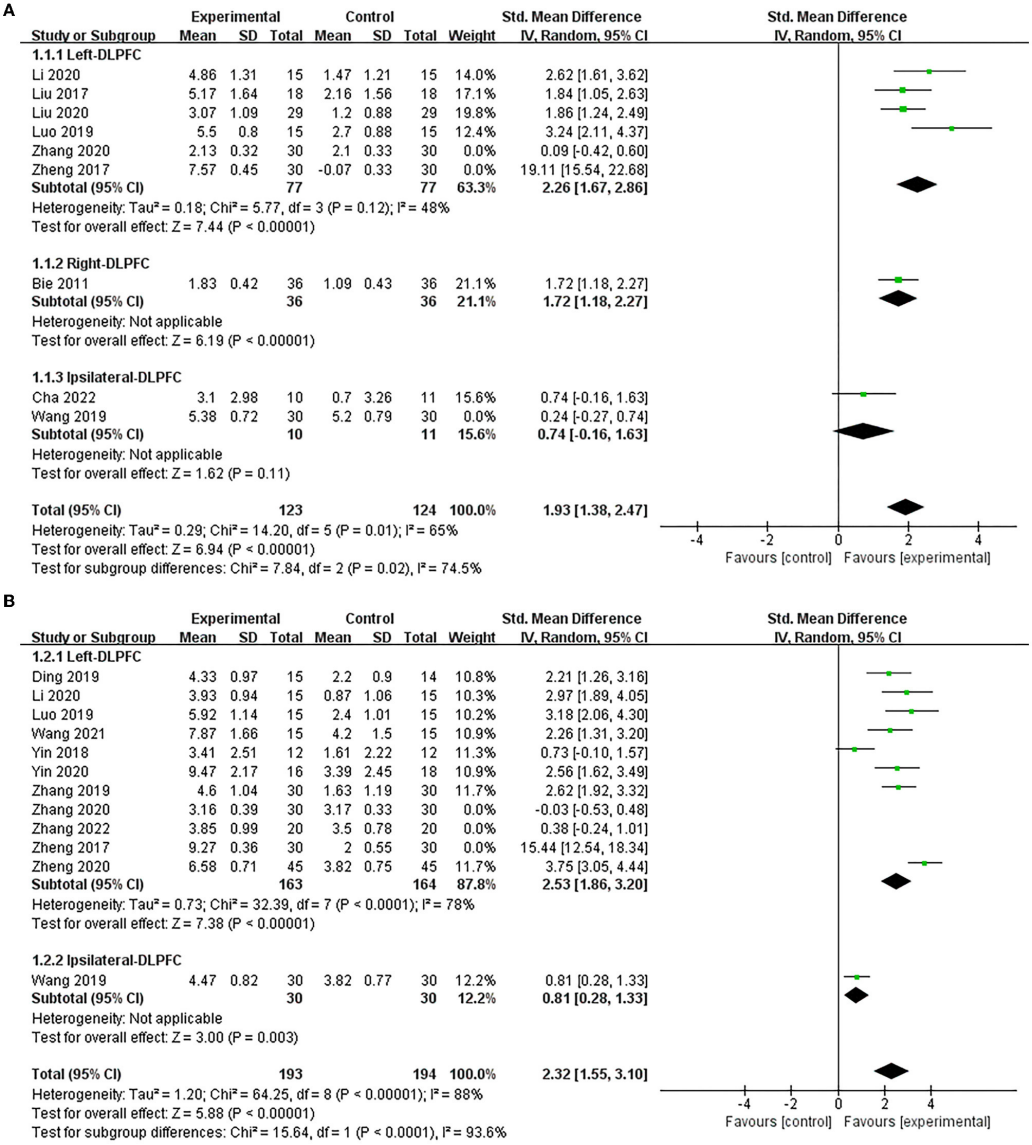
Forest plot of the efficacy of excitatory TMS over the DLPFC on global cognition in patients with PSCI compared to the control group (sensitive analysis). **(A)** MMSE, **(B)** MoCA.

Nine studies (Bie and Wang, 2011; Liu et al., 2017, 2020; Zheng et al., 2017; Luo and Yu, 2019; Wang et al., 2019; Li et al., 2020; Zhang et al., 2020; Cha et al., 2022) used the MMSE to assess the efficacy of excitatory TMS on the global cognitive function in patients with PSCI and showed that the experimental group was significantly improved MMSE scores compared to the control group (SMD = 2.48, 95% CI 1.37–3.59, *P* < 0.0001) (Supplementary Figure 1A). Based on the different stimulation sides and due to higher heterogeneity, we performed subgroup and sensitivity analysis. The results showed that excitatory TMS over the left and right hemispheres DLPFC were both superior to the control group in improving the MMSE scores of the experimental group (SMD = 2.26, 95% CI 1.67–2.86, *P* < 0.00001; vs. SMD = 1.72, 95% CI 1.18–2.27, *P* < 0.00001). However, there was no statistical difference over the ipsilateral DLPFC to stimulation between the two groups (SMD = 0.74, 95% CI −0.16 and 1.63, *P* = 0.11) (Figure 3A).

Twelve studies (Zheng et al., 2017, 2020; Yin et al., 2018, 2020; Ding et al., 2019; Luo and Yu, 2019; Wang et al., 2019, 2021; Zhang and Zou, 2019; Li et al., 2020; Zhang et al., 2020, 2022) used MoCA to assess the efficacy of excitatory TMS on global cognitive function in patients with PSCI and showed that the experimental group demonstrated improved MoCA scores than the control group (SMD = 2.64, 95% CI 1.62–3.66, *P* < 0.00001) (Supplementary Figure 1B). Similarly, based on the different stimulation sides and due to the high heterogeneity, we performed subgroup and sensitivity analysis. Our results showed that excitatory TMS over the left and ipsilateral hemispheres DLPFC were both significantly better than that of the control group in improving the MoCA scores of the experimental group (SMD = 2.53, 95% CI 1.86–3.20, *P* < 0.00001; vs. SMD = 0.81, 95% CI 0.28–1.33, *P* = 0.003) (Figure 3B).

#### 3.4.2. Memory

**Figure 4:**
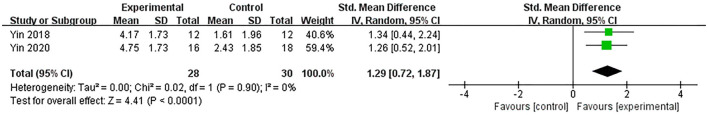
Forest plot of the efficacy of excitatory TMS over the left hemisphere DLPFC on memory in patients with PSCI compared to the control group.

Two studies (Yin et al., 2018, 2020), both using RBMT, assessed the efficacy of excitatory TMS over the left hemisphere DLPFC on memory in patients with PSCI. They showed that the experimental group was significantly superior to the control group in improving memory (SMD = 1.29, 95% CI 0.72–1.87, *P* < 0.0001) (Figure 4).

#### 3.4.3. Attention

**Figure 5:**
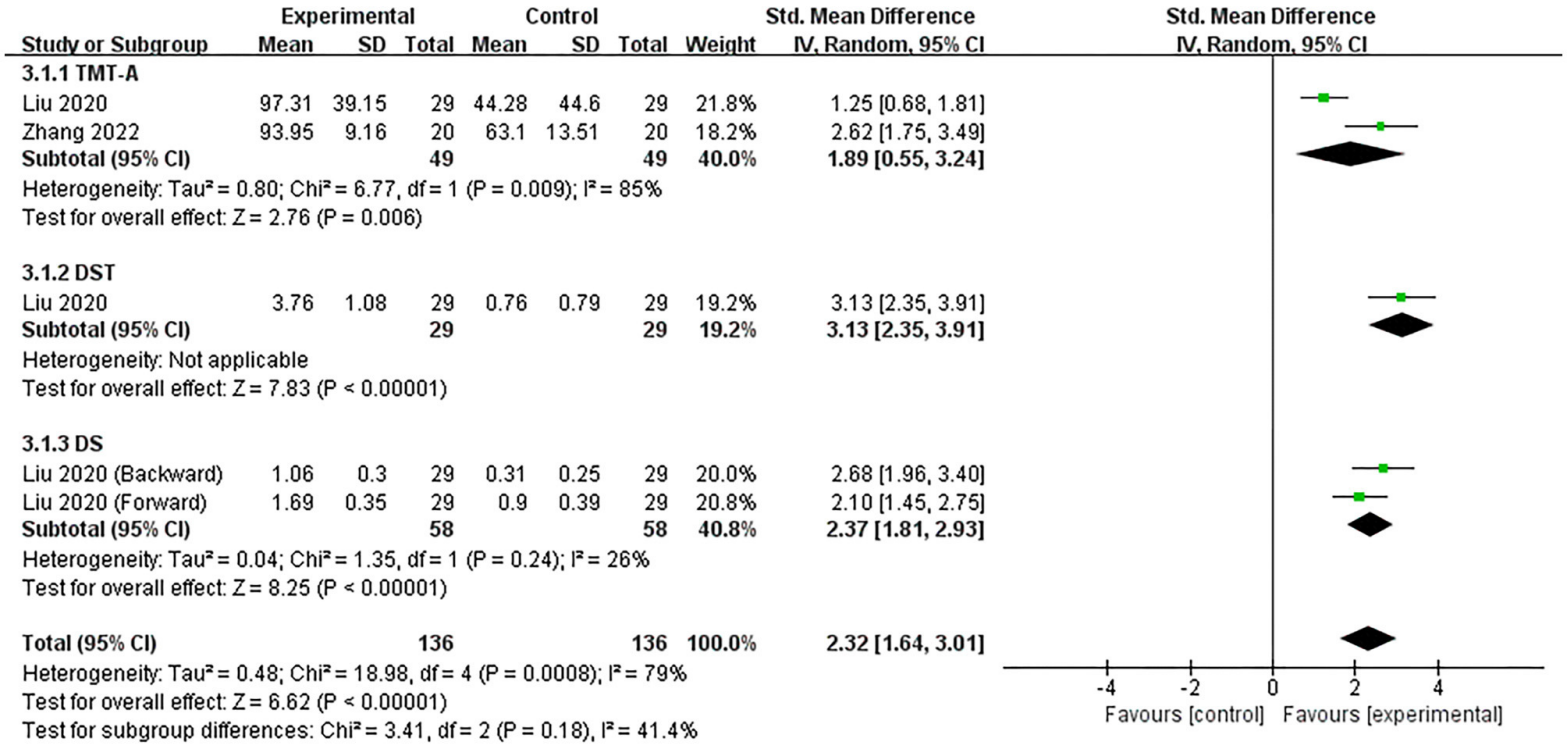
Forest plot of the efficacy of excitatory TMS over the left hemisphere DLPFC on attention in patients with PSCI compared to the control group.

Two studies (Liu et al., 2020; Zhang et al., 2022) assessed the efficacy of excitatory TMS over the left hemisphere DLPFC on attention in patients with PSCI. They showed that attention was significantly improved in the experimental group (SMD = 2.32, 95% CI 1.64–3.01, *P* < 0.00001) (Figure 5). Based on different neuropsychological tests, we performed a subgroup analysis. The changes in TMT-A, DST and DS scores showed that the experimental group were both significantly better than the control group in improving patients' attention intensity and durability, attention conversion, and auditory attention (SMD = 1.89, 95% CI 0.55–3.24, *P* = 0.006; vs. SMD = 3.13, 95% CI 2.35–3.91, *P* < 0.00001; vs. SMD = 2.37, 95% CI 1.81–2.93, *P* < 0.00001).

#### 3.4.4. Execution

**Figure 6:**
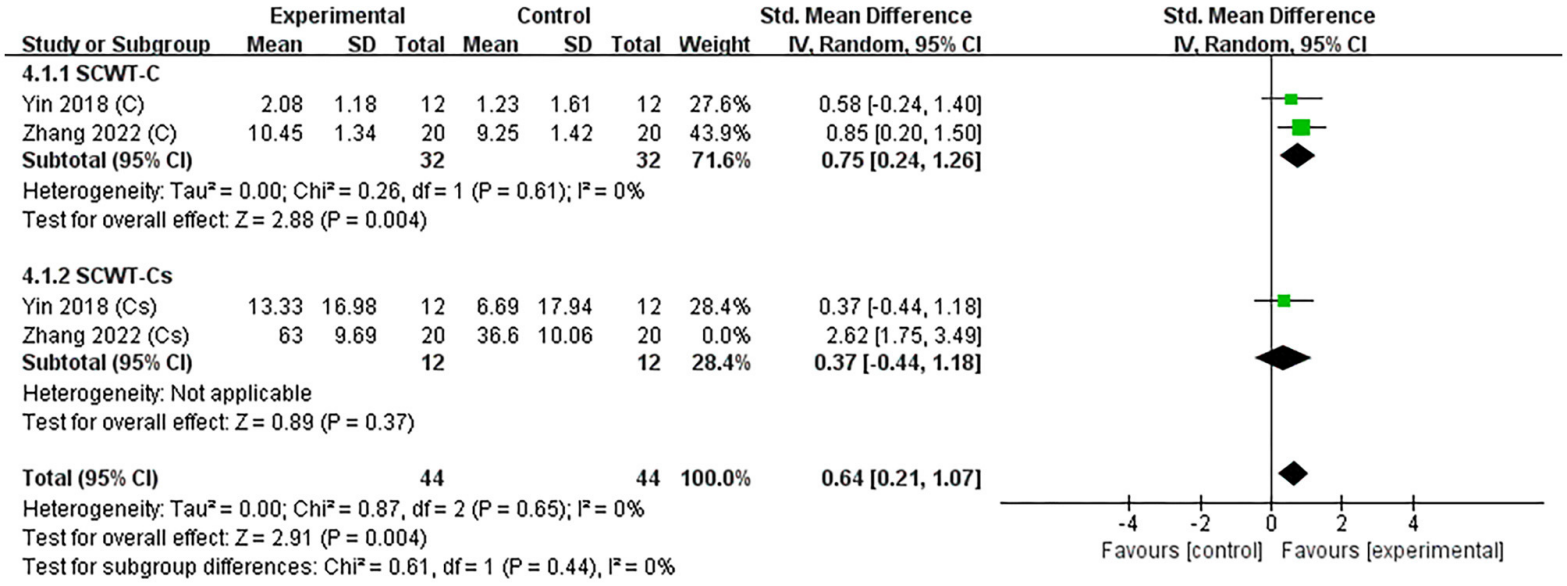
Forest plot of the efficacy of excitatory TMS over the left hemisphere DLPFC on execution in patients with PSCI compared to the control group (sensitive analysis).

Two studies (Yin et al., 2018; Zhang et al., 2022) assessed the efficacy of excitatory TMS over the left hemisphere DLPFC on execution in patients with PSCI. Notably, we extracted the time-consuming and correct numbers for completing the Stroop-C section in the included studies to perform subgroup analysis. The results showed that the experimental group was significantly better than the control group in improving the number of corrects (SMD = 0.75, 95% CI 0.24–1.26, *P* = 0.004), but not statistically different in improving the time-consuming (SMD = 1.49, 95% CI −0.72 to 3.70, *P* = 0.19) (Supplementary Figure 2). Subsequently, we performed a sensitivity analysis to reduce heterogeneity, which decreased only after excluding one neuropsychological test, and the overall effect results on executive function remain unchanged (SMD = 0.64, 95% CI 0.21–1.07, *P* = 0.004) (Figure 6).

#### 3.4.5. Activities of daily living

**Figure 7:**
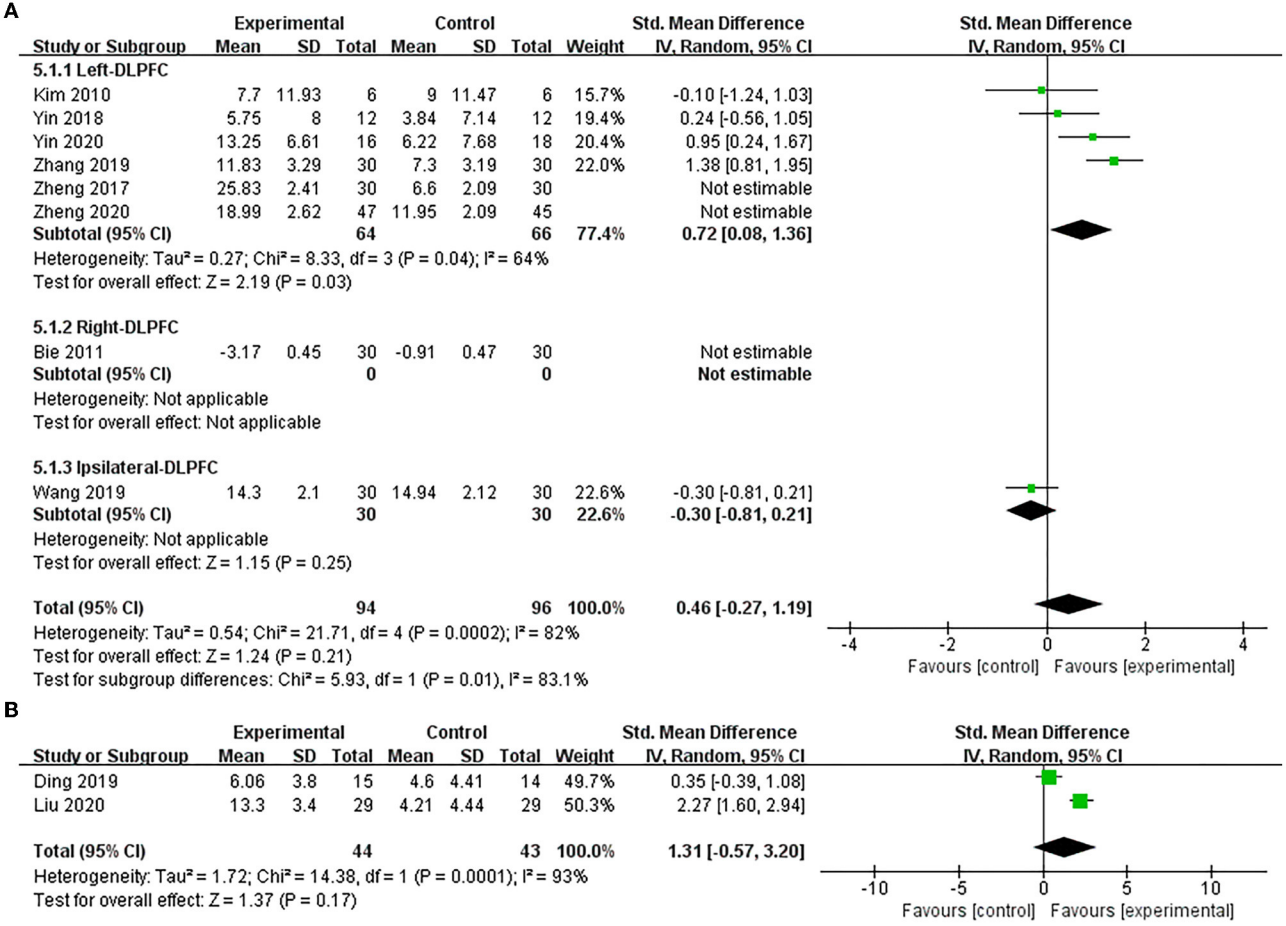
Forest plot of the efficacy of excitatory TMS over the DLPFC on ADL in patients with PSCI compared to the control group. **(A)** MBI (sensitive analysis), **(B)** FIM.

Eight studies (Kim et al., 2010; Bie and Wang, 2011; Zheng et al., 2017, 2020; Yin et al., 2018, 2020; Wang et al., 2019; Zhang and Zou, 2019) used MBI to assess the efficacy of excitatory TMS on ADL in patients with PSCI, and the result showed no statistical difference in improving MBI scores between the two groups (SMD = 1.02, 95% CI −0.63 to 2.68, *P* = 0.22) (Supplementary Figure 3). Based on the different stimulation sides and due to the higher heterogeneity, we performed subgroup and sensitivity analysis. We found that studies of stimulation over the right hemisphere DLPFC were an essential factor contributing to the high heterogeneity of the overall effect. Therefore, we pooled the effect after exclusion. Our results showed that excitatory TMS over the left hemisphere DLPFC was better than that of the control group in improving the MBI scores of the experimental group (SMD = 0.72, 95% CI 0.08–1.36, *P* = 0.03). However, there was no statistical difference over the ipsilateral hemisphere DLPFC to stimulation between the two groups (SMD = −0.30, 95% CI −0.81 to 0.21, *P* = 0.25). Furthermore, the pooled overall effect was not changed (SMD = 0.46, 95% CI −0.27 to 1.19, *P* = 0.21) (Figure 7A).

Only two studies (Ding et al., 2019; Liu et al., 2020) used FIM to assess the efficacy of excitatory TMS over the left hemisphere DLPFC on ADL in patients with PSCI. The results also showed no significant difference between the two groups in improving FIM scores (SMD = 1.31, 95% CI −0.57 to 3.20, *P* = 0.17) (Figure 7B).

#### 3.4.6. P300

**Figure 8:**
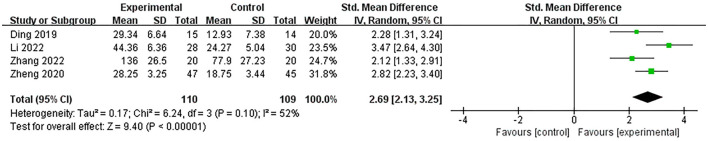
Forest plot of the efficacy of excitatory TMS over the left hemisphere DLPFC on P300 latency in patients with PSCI compared to the control group.

Four studies (Ding et al., 2019; Zheng et al., 2020; Li et al., 2022; Zhang et al., 2022) used the P300 latency to assess the efficacy of excitatory TMS over the left hemisphere DLPFC on cognition in patients with PSCI. The results showed that with acceptable heterogeneity (*P* = 0.10, *I*^2^ = 52%), the experimental group was much better than the control group in improving P300 latency (SMD = 2.69, 95% CI 2.13–3.25, *P* < 0.00001) (Figure 8). Additionally, two studies (Zheng et al., 2020; Li et al., 2022) used P300 amplitude to assess the efficacy of excitatory TMS. However, we did not perform a meta-analysis because the data were not fully available.

#### 3.4.7. Depression

**Figure 9:**
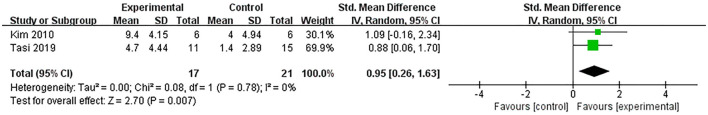
Forest plot of the efficacy of excitatory TMS over the left hemisphere DLPFC on depression in patients with PSCI compared to the control group.

Two studies (Kim et al., 2010; Tsai et al., 2020) used BDI to assess the efficacy of excitatory TMS over the left hemisphere DLPFC on depression in patients with PSCI. They showed that the experimental group was superior to the control group in improving depression (SMD = 0.95, 95% CI 0.26–1.63, *P* = 0.007) (Figure 9).

#### 3.4.8. Adverse events

Of the 19 studies included in this meta-analysis, 8 studies did not mention adverse events (Bie and Wang, 2011; Liu et al., 2017; Ding et al., 2019; Luo and Yu, 2019; Yin et al., 2020; Zhang et al., 2020, 2022; Wang et al., 2021), and 5 reported no adverse events (Kim et al., 2010; Zhang and Zou, 2019; Liu et al., 2020; Tsai et al., 2020; Cha et al., 2022). Five studies reported no obvious adverse events, of which three reported transient mild dizziness or headache, but all were tolerable and relieved with rest (Yin et al., 2018; Li et al., 2020; Zheng et al., 2020); one study reported stimulus-related sneezing symptoms (Li et al., 2022); one study reported patients with inattention or sleep disorders (Zheng et al., 2020). Only one reported the occurrence of seizures in patients (Wang et al., 2019). Thus, more extensive randomized controlled trials are needed to further confirm the efficacy and safety of TMS for PSCI in the future.

## 4. Discussion

Unlike previous studies, this is the first meta-analysis to explore the effects of excitatory TMS over the DLPFC in different cerebral hemispheres on cognitive function in patients with PSCI. Our results showed that excitatory TMS over the left hemisphere DLPFC significantly improved global cognitive function, memory, attention, executive, P300 latency, and depression in patients with PSCI. Additionally, it provided an evidence-based rationale for its clinical application.

As a non-invasive, painless, and safe neuromodulation technique, TMS is based on the principle of electromagnetic induction, where a stored energy capacitor rapidly discharges into the stimulation coil to generate a pulsed magnetic field. In doing so, it creates a painless current to stimulate neurons while affecting the neural activity and cortical excitability in the brain (Rossi et al., 2009). Our study focused on excitatory TMS, including HF-rTMS and iTBS. ITBS is an optimized mode of rTMS with the advantages of low stimulation intensity, short cycle, and long benefit (Pinto et al., 2021). Importantly, the results showed that excitatory TMS improved the cognitive function of patients with PSCI. This is consistent with the findings of Selingardi et al. (2019), which showed that excitatory TMS could promote local nerve regeneration, enhance neuroplasticity and intercortical connectivity, and thus improve cognitive function.

The DLPFC, a common target brain region for TMS research and application, involves various cognitive functions such as memory, attention, and execution (Baker et al., 2014; Panikratova et al., 2020). Consistent with the results of our study, Tsai et al. (2020) found that iTBS intervention over the left hemisphere DLPFC improved the global cognition and memory function of stroke patients. On this basis, this study further performed subgroup analysis and, for the first time, explored the efficacy difference of excitatory TMS over the DLPFC in different hemispheres to improve cognitive function, ADL, and depression. The results showed that the left hemispheric DLPFC was a more effective treatment area than excitatory TMS treatment on the ipsilateral and right hemispheres DLPFC. The left hemisphere DLPFC, a key node of the central executive network (CEN) (Bigliassi and Filho, 2022), is closely related to advanced cognitive functions such as working memory, episodic memory, and selective attention. Furthermore, studies have shown that excitatory TMS over the left hemisphere DLPFC can improve cognition in patients by promoting corticospinal excitability (Guse et al., 2010; Li et al., 2020). Motes et al. (2018) also observed by functional magnetic resonance imaging (fMRI) that the improvement in cognitive function was strongly correlated with enhanced neural activity over the left hemisphere DLPFC.

Following a stroke, patients often have changes in brain tissue structure due to insufficient blood and oxygen supply to the brain (D'Souza et al., 2021), gradual degeneration of brain nerves with neuronal loss, and damage to the conduction pathways of neurotransmitters such as acetylcholine, causing impairment in brain cell information transmission, which gradually manifests as cognitive impairment (Girouard and Iadecola, 2006). Our study showed that excitatory TMS over the left hemisphere DLPFC significantly improved memory, attention, executive, and global cognitive function in patients with PSCI. Notably, the improvement of cognitive function can be attributed to multiple factors. First, excitatory TMS can reduce the inhibitory control of pyramidal cells to increase excitatory output (Cirillo et al., 2017), increase cerebral blood flow, improve brain cell metabolism, promote white matter repair and growth, and thereby repair cognitive circuits (Wu et al., 2021). Second, cognitive function is improved by binding DLPFC to the caudate nucleus, promoting the expression of neurotrophic factors and increasing the release of neurotransmitters (Anderkova and Rektorova, 2014; Hoy et al., 2016). Third, the left hemisphere DLPFC contains the vital cognitive function network (Gomes-Osman et al., 2018), excitatory TMS over the left hemisphere DLPFC can also increase cortical excitability and neuroplasticity by inducing long-term potentiation (LTP) (Wang and Voss, 2015), as well as modulating functional connectivity between brain networks (Yang et al., 2015a). Li et al. (2020) used fMRI to demonstrate that rTMS improved neuroplasticity and changes in neural activity, enhancing the functional connection between the target area and other cognitive processing networks. Furthermore, excitatory TMS can promote hippocampal cells' proliferation and neural regeneration in the dentate gyrus, which is closely related to memory and learning processes (Ueyama et al., 2011).

P300, an objective electrophysiological index, reflects the information processing of working memory and the speedy processing of participating in decision-making (Dejanović et al., 2015). Notably, latency is related to the information processing of the external environment, reflecting the speed at which the brain classifies and recognizes external stimuli and representing the degree of excitement of the central nervous system during information recognition and processing (Rêgo et al., 2012). Our study showed that excitatory TMS over the left hemisphere DLPFC significantly shortened the P300 latency and improved the global cognitive function in patients with PSCI. This is consistent with previous studies (Pinto et al., 2021), where these improvements may be related to the iTBS-mediated enhancement of neurotransmitter dopaminergic and glutamatergic connections (Anderkova and Rektorova, 2014). Our study also found that excitatory TMS had no apparent therapeutic effect in improving ADL, which was inconsistent with the study of Li et al. (2021b). This may be attributed to the heterogeneity of stimulation protocols between studies. Furthermore, in an animal experiment in rats, HF-rTMS and iTBS on the motor cortex effectively promoted neural regeneration and increased cortical excitability (Luo et al., 2017). This study also reported that excitatory TMS over the left hemisphere DLPFC improved depression in patients with PSCI. However, the effectiveness of HF-rTMS and iTBS in treating depression in patients with PSCI remains controversial in previous studies (De Risio et al., 2020; Cash et al., 2021). Therefore, it is necessary to explore excitatory TMS' efficacy further and study its mechanism in future studies.

### 4.1. Limitations

Our study also has some noted limitations. First, we performed subgroup analysis based on the different stimulus areas, but only one study was included in some subgroups. Thus, this may lead to a certain bias in the results. Second, due to the limited number of included studies, we cannot perform subgroup analysis on stroke type and TMS stimulation parameters. Third, our study focuses on the immediate effects after excitatory TMS treatment and lacks studies on the long-term effects.

## 5. Conclusions

Previous literature lacks research on the effect of excitatory TMS over the DLPFC in different hemispheres on the rehabilitation outcome of PSCI patients. This meta-analysis found that compared to other hemispheric sides, excitatory TMS over the left hemisphere DLPFC was a more effective stimulation area, which can significantly improve global cognitive function, memory, attention, executive, P300 latency, and depression in patients with PSCI. However, there was no apparent treatment effect on improving ADL. In the future, more randomized controlled trials with large-sample, high-quality, and follow-up are needed to explore a usable protocol further.

## Data availability statement

The original contributions presented in the study are included in the article/Supplementary material, further inquiries can be directed to the corresponding author.

## Author contributions

KH, JL, ZT, and WS participated in literature search and screening, quality assessment, and data extraction. KH and YL performed data collation and analysis. KH wrote the manuscript. HZ and HL participated in the study design and guidance and reviewed the final manuscript. All authors contributed to the article and approved the submitted version.
