# Supplementary material for: Effects of excitatory transcranial magnetic stimulation over the different cerebral hemispheres dorsolateral prefrontal cortex for post-stroke cognitive impairment: a systematic review and meta-analysis
Source: Front Neurosci. 2023 May 16;17:1102311. doi: 10.3389/fnins.2023.1102311 (PMC10228699; doi:10.3389/fnins.2023.1102311)
Supplement: Supplementary file 1 [file Data_Sheet_1.docx]

Supplementary Material

**1 Supplementary Tables**

Table S1 | Supplementary search strategy

| Number | Search strategy |
| --- | --- |
| #1 | TS = (“TMS” OR “rTMS” OR “Transcranial Magnetic Stimulation*” OR “Magnetic Stimulation*,Transcranial” OR “Stimulation*, Transcranial Magnetic” OR “Repetitive transcranial magnetic stimulation*” OR “Transcranial Magnetic Stimulation*, Repetitive” OR “TBS” OR “iTBS” OR “theta burst stimulation*” OR “θ burst stimulation*” OR “cTBS” OR “Continuous transcranial burst stimulation*” OR “Transcranial Magnetic Stimulation*, Single Pulse” OR “Transcranial Magnetic Stimulation*, Paired Pulse”) |
| #2 | TS = (“Cognition*” OR Neurocogniti* OR “Cognitive Function*” OR “Function*, Cognitive” OR “Cognitive impairment*” OR “Cognitive dysfunction*” OR “memory” OR “attention*” OR “Executive Function*” OR “Executive Control*” OR “calculati*” OR “unilateral neglect*” OR “Problem Solving”) |
| #3 | TS = (“Stroke*” OR “Cerebrovascular Accident*” OR “CVA” OR “CVAs” OR “Apoplexy” OR “Cerebrovascular Apoplexy” OR “Apoplexy, Cerebrovascula” OR “Cerebrovascular Stroke*” OR “Stroke*,Cerebrovascular” OR “Brain Vascular Accident*” OR “Vascular Accident*, Brain” OR “Cerebral Stroke*” OR “Stroke*, Cerebral” OR “Stroke*, Acute” OR “Acute Stroke*” OR “Cerebrovascular Accident*, Acute” OR “Acute Cerebrovascular Accident*” ) |
| #4 | #1 and #2 and #3 |

Table S2 | Characteristics of included studies

| **Study** | **Country** | **Population**  **diagnosis** | **Sample**  **size**  **(E/C)** | **Men**  **(%, E/C)** | **age**  **(year, E/C)** | **Onset**  **time**  **(E/C)** | **Education**  **(y , E/C)** | **Intervention design** | | **Stimulation condition** | | **Outcome measure** | **Follow-up**  **(month)** | **Drop out**  **(E/C)** | **PEDro**  **score** |
| --- | --- | --- | --- | --- | --- | --- | --- | --- | --- | --- | --- | --- | --- | --- | --- |
|  |  |  |  |  |  |  |  | **E** | **C** | **Site**  **(DLPFC)** | **Protocol** |  |  |  |  |
| Cha  et al.  (2022) | South korea | PSCI | 10/11 | 80.0/  72.7 | 53.80 ± 8.20  61.80 ± 9.70 | 29.50 ± 49.50 months  18.30 ± 24.60 months | 10.40 ± 3.80  12.20 ± 2.30 | HF-rTMS | Sham | Ipsilateral | 8 coil, 100% RMT, 20 Hz, 2000 pulses, 20 min/d,  5d /week, 2 weeks | ①Global Cognition: MMSE | 3 | 0/0 | 6 |
| Li  et al.  (2022) | China | PSCI | 28/30 | 53.3/  60.0 | 69.5 (60.0,78.0) 66.0 (53.0,75.0) | 25.0 (17.0, 30.0) days  25.0 (18.0, 30.0) days | Illiteracy:4/6  primary school:12/10  Junior high school and above:12/14 | iTBS  +CR | Sham  +CR | Left | 8 coil, 100% RMT, 600 pulses,  192 s/d, 5 d /week, 2 weeks | ①Global Cognition: P300 | NR | 2/0 | 10 |
| Zhang  et al.  (2022) | China | PSCI | 20/20 | 55.0/  40.0 | 54.00 ± 7.00  57.00 ± 6.00 | 6.00 ± 2.00 months  6.00 ± 2.00 months | 12.00 ± 3.00  11.00 ± 2.00 | HF-rTMS  +CR | Sham  +CR | Left | 80% RMT, 5Hz, 3000 pulses, 20 min/d, 5 d/ week, 4 weeks | ①Global Cognition: MoCA、P300  ②Attention: TMT  ③Execution: SCWT | NR | 0/0 | 7 |
| Wang et al.  (2021) | china | PSCI | 15/15 | 73.3/  66.7 | 57.80 ± 13.00  58.80 ± 9.30 | 52.50 ± 21.60 days  57.03 ± 18.50 days | 9.40 ± 3.60  8.80 ± 3.60 | HF-rTMS  +CR | Sham  +CR | Ipsilateral | 8 coil, 80% RMT, 10 Hz,  20 min/d, 5 d/ week, 8 weeks | ①Global Cognition: MoCA | NR | 0/0 | 9 |
| Liu  et al.  (2020) | China | PSAI | 29/29 | 34.5/  65.5 | 58.55 ± 6.24  57.69 ± 7.25 | 8.79 ± 1.84 months  8.62 ± 1.84 months | 9.76 ± 2.80  8.55 ± 3.32 | HF-rTMS  +CR | Sham  +CR | Left | 8 coil, 90% RMT, 700 pulses,  5 d /week, 4 weeks | ①Global Cognition: MMSE  ②Memory: DST、DS  ③Attention: TMT  ④ADL: FIM | NR | 2/2 | 10 |
| Yin  et al.  (2020) | China | PSCI | 16/18 | 87.5/  88.9 | 56.69 ± 12.92  58.17 ± 11.27 | 52 (38.25, 98.75)days  55 (39.75, 94.75)days | 10.03 ± 4.15  9.33 ± 3.87 | HF-rTMS  +CR | Sham  +CR | left | 80% RMT, 10 Hz, 2000 pulses, 20 min/d, 5 d/ week,  4 weeks | ①Global Cognition: MoCA  ②Memory: RBMT  ③Execution: VST  ④ADL: MBI | NR | 2/0 | 8 |
| Li et al.  (2020) | China | PSCI | 15/15 | 46.7/  60.0 | 65.47 ± 3.68  64.53 ± 4.72 | 22.73 ± 8.05 days  19.13 ± 7.95 days | 9.20 ± 2.31  9.07 ± 2.63 | HF-rTMS  +CR | Sham  +CR | Left | 8 coil, 100% RMT, 5 Hz, 2000 pulses, 5 d/ week, 3 weeks | ①Global Cognition:  MMSE、MoCA | ­­NR | 0/0 | 9 |
| Zhang  et al.  (2020) | China | PSVCI | 30/30 | 63.3/  53.3 | 49.07 ± 9.26  49.17 ± 10.58 | 49.73 ± 28. 83 days  51.07 ± 28. 69 days | 12.27 ± 2.92  13.16 ± 3.37 | HF-rTMS  +CR | CR | Left | 8 coil, 80% RMT, 15 Hz, 3000 pulses, 20 min/d, 5 d/ week,  8 weeks | ①Global Cognition:  MMSE、MoCA | ­­NR | 0/0 | 6 |
| Zheng  et al.  (2020) | China | PSCI | 55/51 | 65.5/  64.7 | 58.30 ±7.90  59.70 ±6.30 | 48.7 ± 14.4 days  47.3 ± 11.8 days | 9.50 ±3.30  9.80 ±3.10 | HF-rTMS  +DT | Sham  +DT | Left | 8 coil, 80% RMT, 10 Hz,  20 min/d, 5 d/ week, 4weeks | ①Global Cognition: MoCA、P300  ②Memory: RBMT  ③ADL: MBI | ­­NR | 0/0 | 9 |
| Tsai  et al. (2010) | China | PSCI | 11/15 | 81.8/  86.7 | 57.45 ± 12.30  56.23 ±12.00 | 33.27 ± 26.40 months  38.00 ± 7.90 months | 14.00 ± 2.80  13.64 ± 1.90 | HF-rTMS | Sham | Left | 8 coil, 80% RMT, 5 Hz, 600 pulses, 5 d/ week, 2 weeks | ①Cognition: RBANS  ②Depression: BDI | ­­NR | 3/0 | 10 |
| Luo  et al.  (2019) | China | PSCI | 15/15 | 46.7/  60.0 | 65.80 ± 3.30  64.30 ± 5.10 | 30.50 ± 7.80 days  32.90 ± 4.40 days | NR | HF-rTMS  +CR | CR | Left | 8 coil, 80-120% RMT, 5 Hz, 1050 pulses, 20 min/d,  5 d/ week, 3 weeks | ①Global Cognition:  MMSE、MoCA | ­­NR | 0/0 | 9 |
| Ding  et al.  (2019) | China | PSCI | 15/14 | 73.3/  64.3 | 53.67 ± 7.58  53.53 ± 7.65 | 7.60 ± 2.58 weeks  7.07 ± 2.12 weeks | 7.20 ± 4.31  7.13 ± 4.05 | HF-rTMS  +CR | Sham  +CR | Ipsilateral | circular coil, 110% RMT,  5 Hz, 20 min/d, 6 d/ week,  2 weeks | ①Global Cognition: MoCA、P300  ②ADL: FIM | 2 | 0/1 | 9 |
| Zhang  et al.  (2019) | China | PSCI | 30/30 | 66.7/  60.0 | 58.44 ± 16.60  55.11 ± 18.03 | 46.83 ± 28,13 days  49.00 ±37.01 days | 11.89 ± 4.10  11.61 ± 2.75 | HF-rTMS  +CR | Sham  +CR | Left | 8 coil, 80% RMT, 5 Hz, 1600 pulses, 20 min/d, 5 d/ week,  4 weeks | ①Global Cognition: MoCA、P300  ②ADL: MBI | NR | 0/0 | 9 |
| Wang  et al.  (2019) | China | PSCI | 30/30 | 73.3/  70.0 | 54.57 ± 5.83  56.13 ± 6.81 | 8.23 ± 2.53 weeks  8.60 ± 2.63 weeks | 12.07 ± 3.45  11.27 ± 3.16 | HF-rTMS+CR | CR | Ipsilateral | 8 coil, 80-120% RMT, 5~10 Hz, 600~800 pulses, 20 min/d, 5 d/ week, 4 weeks | ①Global Cognition:  MMSE、MoCA  ②ADL: MBI | NR | 0/0 | 7 |
| Yin  et al.  (2018) | China | PSCI | 12/13 | 91.7/  92.3 | 58.58 ± 11.98  60.15 ± 10.29 | 59.83 ± 30.59 days  56.15 ± 23.74 days | 9.54±3.24  8.92±3.57 | HF-rTMS  +CR | Sham  +CR | Left | 8 coil, 80% RMT, 10 Hz, 2000 pulses, 20 min/d, 5 d/ week,  4 weeks | ①Global Cognition: MoCA  ②Memory: RBMT  ③Execution: VST  ④ADL: MBI | NR | 0/0 | 9 |
| Liu  et al.  (2017) | China | PSEI | 18/18 | 61.1/  50.0 | 65.33 ± 7.05  62.61 ± 9.98 | 8.44 ± 1.97 momths  9.33 ± 1.64 momths | 7.56 ± 3.81  9.50 ± 4.88 | HF-rTMS+CR | Sham  +CR | Left | 8 coil, 90% RMT, 10 Hz, 700 pulses, 10.5 min/d, 5 d/ week,  4 weeks | ①Global Cognition: MMSE  ②Memory: DST、DS  ③Attention: WCST | NR | 0/0 | 9 |
| Zheng  et al.  (2017) | China | PSVCI | 30/30 | 46.7/  56.7 | 58.80 ± 13.54  61.97 ± 11.39 | 2.73 ± 1.26 months  3.00 ± 1.11 months | primary school: 6/7  Junior high school: 15/14  univ.: 9/9 | HF-rTMS  +CR | CR | Left | 8 coil, 80% RMT, 20 Hz, 1500 pulses, 20 min/d, 5 d/ week,  6 weeks | ①Global Cognition:  MMSE、MoCA  ②ADL: MBI | NR | 0/0 | 8 |
| Bie  et al.  (2011) | China | PSMCI | 36/36 | 55.6/  50.0 | 61.40 ± 9.80  62.10 ± 10.20 | 86.00 days  88.00 days | NR | HF-rTMS  +AR | Sham  +AR | Right | circular coil, 80% RMT, 10 Hz, 20 min/d, 5 d/ week,  2 weeks | ①Global Cognition: MMSE  ②ADL: MBI | 2.5 | 0/0 | 8 |
| Kim  et al.  (2010) | Korea | PSCI | 6/6 | 66.7/  66.7 | 53.50 ± 16.90  66.80 ± 17.20 | 241.20 ± 42.50 days  69.70 ± 39.00 days | NR | HF-rTMS | Sham | Left | 8 coil, 80% RMT, 10 Hz,  450 pulses, 5 d/ week,  2 weeks | ①Global Cognition: MMSE  ②ADL: MBI  ③Depresson: BDI | NR | 0/0 | 10 |

Note: Data were expressed as mean ± SD or median (interquartile range [IQR]).

Abbreviations: E, experimental group; C, control group; DLPFC, dorsolateral prefrontal cortex; PSCI, post-stroke cognitive impairment; PSMCI, post-stroke mild cognitive impairment; PSVCI, post-stroke vascular cognitive impairment; PSEI, post-stroke executive impairment; PSAI, post-stroke attention impairment; HF-rTMS, high-frequency repetitive transcranial magnetic stimulation; iTBS, intermittent theta burst Stimulation; CR, conventional rehabilitation; AT, acupuncture therapy; DT, donepezil therapy; RMT, resting motor threshold; MMSE, mini-mental state examination; MoCA, montreal cognitive assessment; RBMT, rivermead behavior memory test; DST, digit symbol test; DS, digital span test; RBANS, repeatable battery for the assessment of neuropsychological status; TMT, trail making test; SCWT, stroop color word test; VST, victoria stroop test; WCST, wsiconsin card sorting test; MBI, modified barthel index; FIM, functional independence measure; P300, P300 from event-related potential measurements; BDI, Beck Depression Inventory; NR, not reported；

**2 Supplementary Figures**

FIGURE S1 | Forest plot of the efficacy of excitatory TMS over the DLPFC on global cognition in patients with PSCI compared to the control group. (A) MMSE, (B) MoCA.

FIGURE S2 | Forest plot of the efficacy of excitatory TMS over the left hemisphere DLPFC on execution in patients with PSCI compared to the control group.

FIGURE S3 | Forest plot of the efficacy of excitatory TMS over the DLPFC on MBI in patients with PSCI compared to the control group.
