# Supplementary figures and images for: Effects of excitatory transcranial magnetic stimulation over the different cerebral hemispheres dorsolateral prefrontal cortex for post-stroke cognitive impairment: a systematic review and meta-analysis
Source: Front Neurosci. 2023 May 16;17:1102311. doi: 10.3389/fnins.2023.1102311 (PMC10228699; doi:10.3389/fnins.2023.1102311)

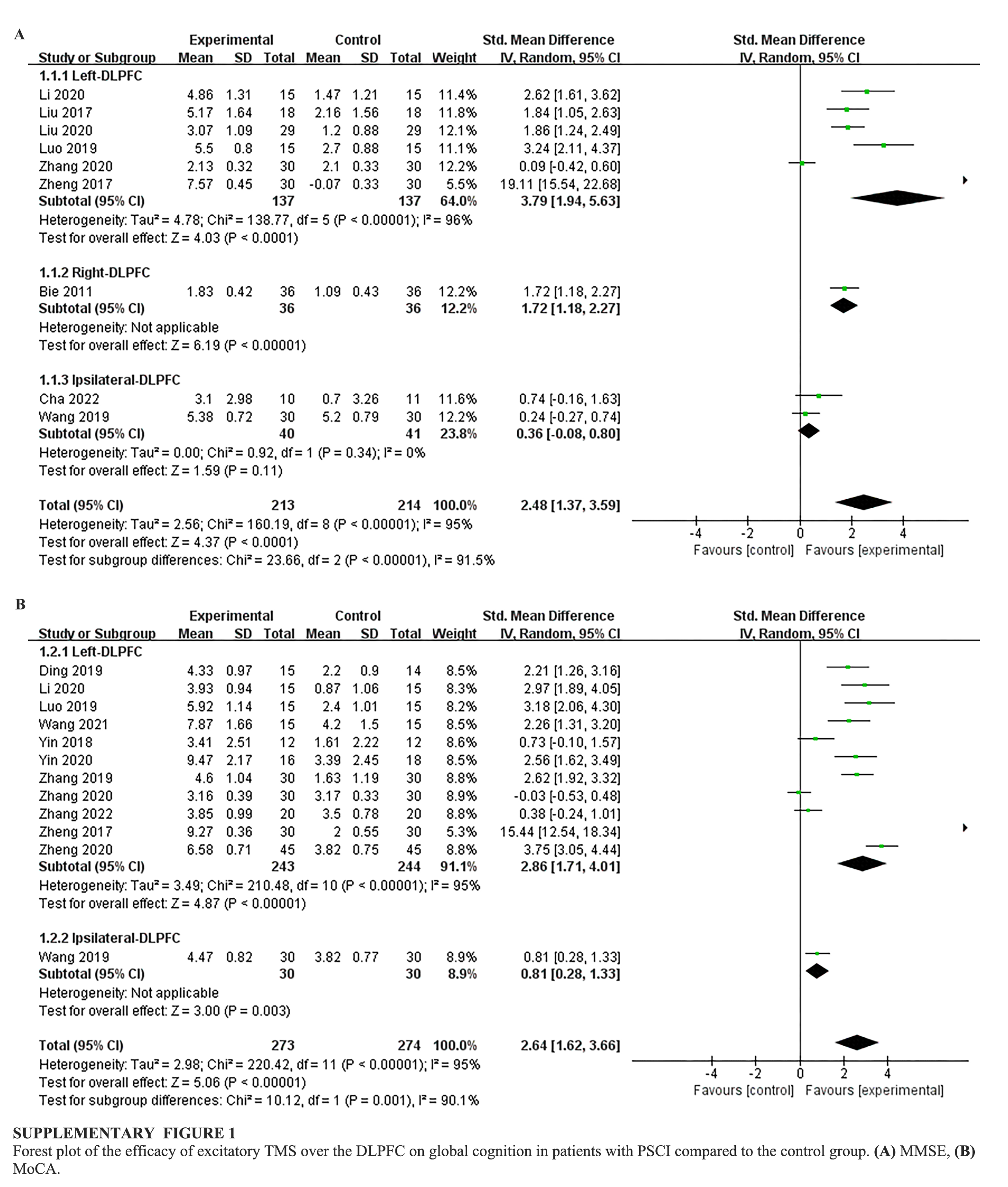

Supplement: Supplementary file 2 [file Image_1.TIF]

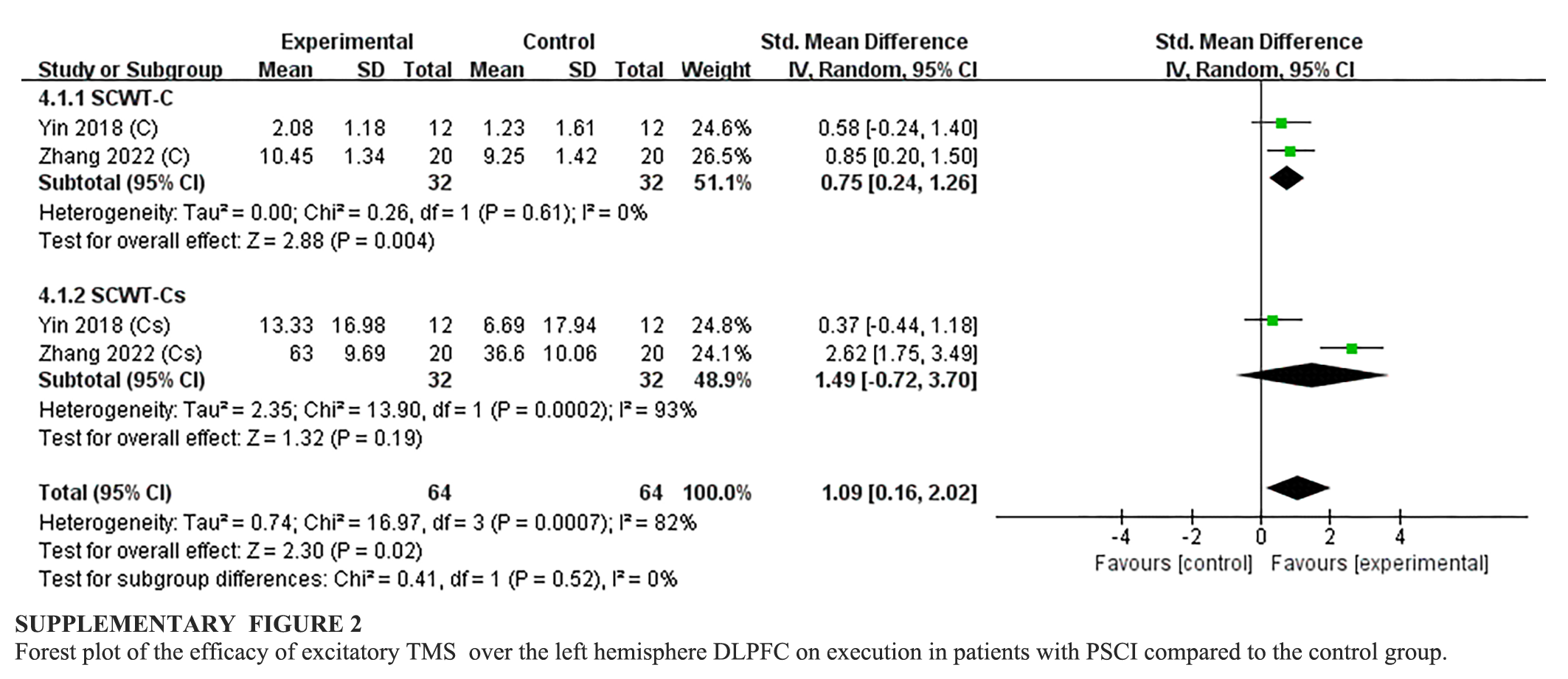

Supplement: Supplementary file 3 [file Image_2.TIF]

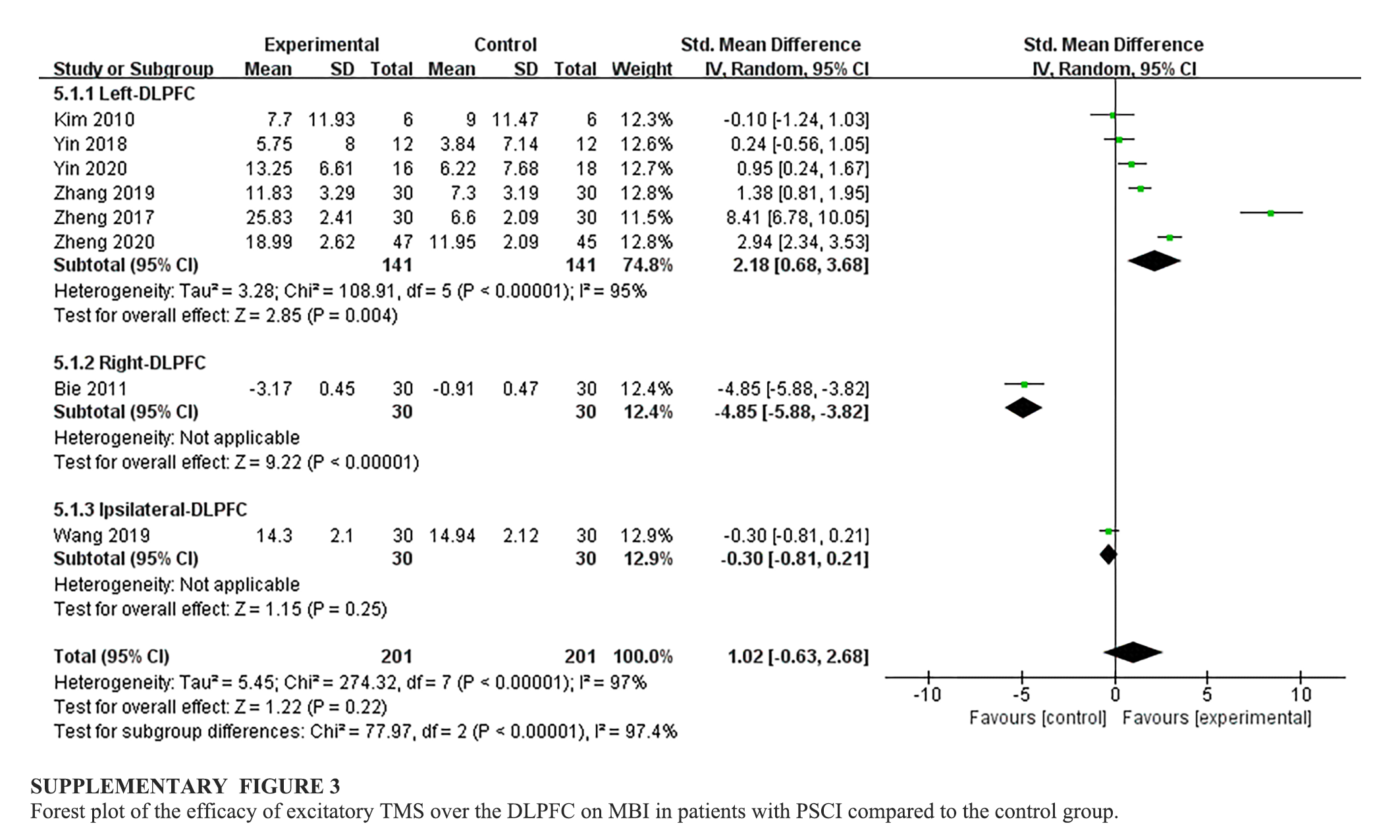

Supplement: Supplementary file 4 [file Image_3.TIF]
